# Lumbar Spondylodiscitis Caused by* Burkholderia cepacia* in a Previously Healthy Patient

**DOI:** 10.1155/2017/1396950

**Published:** 2017-10-15

**Authors:** Danielle Jaafar, Maroun Rizkallah, Firas Atallah, Falah Bachour, Angelique Barakat, Ghassan Maalouf, Matta Matta

**Affiliations:** ^1^Department of Medicine, Hôtel Dieu de France, University Medical Center, Saint-Joseph University, P.O. Box 16683, Achrafieh, Lebanon; ^2^Division of Orthopedic Surgery, Department of Surgery, Bellevue Medical Center, Saint-Joseph University, P.O. Box 16683, Achrafieh, Lebanon; ^3^Division of Orthopedic Surgery, Department of Surgery, Bellevue Medical Center, Saint-Joseph University, P.O. Box 295, Mansourieh, Metn, Lebanon; ^4^Bellevue Medical Center, Saint-Joseph University, P.O. Box 295, Mansourieh, Metn, Lebanon

## Abstract

*Burkholderia cepacia *spondylodiscitis is a rare entity that has been reported in only four cases. We hereby report the case of a 43-year-old, previously healthy, woman who was found to have a* B. cepacia* spondylodiscitis after a cholecystectomy. She was treated with a three-month regimen of ceftazidime and ciprofloxacin with complete clinical, biological, and radiological remission.

## 1. Introduction

Pyogenic spondylodiscitis is a rare infection that usually affects patients older than 50 years with a history of immunodeficiency or nosocomial infection [1]. It is caused by* Staphylococcus aureus* in more than half of the cases [1, 2].* Burkholderia cepacia* spondylodiscitis is a rare entity that has only been reported in four cases in the medical literature. We hereby report the case of a 43-year-old, previously healthy woman, who was found to have a* B. cepacia* spondylodiscitis after a cholecystectomy. She was treated with a three-month regimen of ceftazidime and ciprofloxacin with complete clinical, biological, and radiological remission.

## 2. Case Description

A 43-year-old female Syrian patient, otherwise healthy with no history of drug use, was admitted to a Syrian hospital with a diagnosis of cholecystitis. The day after her cholecystectomy, she started complaining of diffuse myalgia and lower back pain irradiating to her left lower extremity with no fever or chills. She was put on myorelaxants, NSAIDs, and paracetamol for a probable lumbosacral neuropathy and was discharged home on cefepime and amoxicillin/clavulanate for 15 days without any microbiological documentation. One month later, she was referred to a neurologist and a cervical and lumbosacral MRI were performed. These showed degenerative changes with discopathy at the L5-S1 level. An X-ray guided epidural steroid injection was performed and the patient was started on tegretol and a tricyclic antidepressant. Her clinical condition kept on deteriorating. A second lumbar MRI was done on the 26th of July 2014 and showed findings suggestive of spondylodiscitis at the L5-S1 level. Her labs showed an increase in WBC and CRP compatible with infection. Accordingly, she was started on cotrimoxazole and linezolid and then switched to levofloxacin and meropenem due to a lack of improvement. No microbiological identification was made.

Because of the patient's nonresponse to treatment, she decided to seek medical advice at our facility. On physical examination, local tenderness was noted at the level of L5-S1 with left lower extremity paresis and lumbar spine stiffness. An open surgical bone biopsy was done and a specimen is sent for microbiological identification. The deep bone culture confirmed infection with* B. cepacia* sensitive to piperacillin, piperacillin/tazobactam, ceftazidime, cefepime, meropenem, ciprofloxacin, and trimethoprim/sulfamethoxazole. The bacterium was identified by API NF (Biomerieux) # 1477573.

The patient was started on empirical treatment with teicoplanin and imipenem and then switched to ciprofloxacin 400 mg TID and ceftazidime 2 g TID after bacterial identification. At the end of a 3-month course, the patient was pain-free. The control lumbar MRI showed marked improvement of the lesions and her CBC and CRP reached normal levels. She remained asymptomatic on long term follow-up.

## 3. Discussion

Discitis and osteomyelitis are both spinal infections that often occur in conjunction with one another; osteomyelitis and spondylodiscitis are consequently used interchangeably to describe the infection [3]. Pyogenic spondylodiscitis is uncommon with an incidence of 5–5.3 per million patients per year [3]. It mostly affects patients in their 5th decade, at the lumbar level. There is a 2 : 1 unexplained male predominance [1, 3].

Pathogens can reach the spine via three routes: by direct inoculation after a trauma or vertebral surgery, by hematogenous spread after skin, oral, urinary, digestive, or pulmonary infections, or by contiguous spread from surrounding infected tissues. Diabetes mellitus, malignancy, immunosuppression, liver disease, end-stage renal failure, liver cirrhosis, intravenous drug use, previous infection, particularly bloodstream infection, hemodialysis, catheter-related sepsis, and other nosocomial infections or procedures have all been described as risk factors for infectious spondylodiscitis [3].

*Staphylococcus aureus *is the most isolated germ and accounts for more than 50% of the cases. It is followed in incidence by tuberculosis, Gram negative bacilli, and brucellosis in endemic areas and in 10% of cases no germ is isolated [1, 2, 4–6].

As for* B. cepacia* osteomyelitis, it is a very uncommon entity and only four cases were reported in immunocompetent patients. Cervical osteomyelitis was described after rhinoplasty [7] and in an IV drug user [8], thoracic osteomyelitis in a healthy farmer with no surgical history [9], and lumbar osteomyelitis after a fall on an icy road that led to a wound [10].

*Burkholderia cepacia*, formerly called* Pseudomonas cepacia*, is a motile, nonfermenting, aerobic Gram negative rod that is ubiquitous in the environment. It is commonly found in water, soil, and plants and was found to be the cause of the onion rot in the 1950s when it was first described. It is a rapidly growing germ with minimal nutritional needs that can survive for a long time in harsh environments and that is resistant to disinfectants [7, 9, 10].

In humans, it mainly causes respiratory infections in patients with cystic fibrosis [11]. It has been described in bacteremia, endocarditis, wound infection, septic arthritis, osteomyelitis, meningitis, peritonitis, and urinary and respiratory tract infections in immunocompromised patients and intravenous drug users [11]. More and more healthy patients were diagnosed with* B. cepacia* infections in the health care setting which lead to investigations in order to define a possible source. The pathogen was isolated in several medical products such as intravenous fluids, dialysis fluids, ultrasound gels, nebulizers, thermometers, and tap water [7].

*B. cepacia* bacteria have developed resistance to beta-lactams by means of an inducible chromosomal beta-lactamase and altered penicillin-binding proteins [11]. Antibiotic efflux pumps can also lead to resistance to trimethoprim, chloramphenicol, and fluoroquinolones. Antibiotics that are active against* B. cepacia *include meropenem, minocycline, third-generation cephalosporins, and fluoroquinolones [11]. Due to the broad resistance pattern, a combination therapy with a synergy effect is preferred, which includes either ceftazidime or trimethoprim/sulfamethoxazole [10, 11].

A 12-week course of antibiotics was adopted for several reasons. The unusual germ, the residency of the patient in Syria which was war torn with a high risk of medication shortage, and finally the impossibility to have a follow-up at 6 weeks due to the war condition there made us extend the antibiotic therapy course from 6 to 12 weeks. The patient did not have any concomitant surgical treatment, and the last follow-up occurred 6 months after stopping the antibiotics.

Our case report is unusual on two levels. On one hand, it may be considered an atypical presentation of an uncommon pathology because the patient was previously healthy and had none of the pyogenic vertebral osteomyelitis' risk factors cited above. On the other hand,* B. cepacia, *in itself, is a rare human pathogen and is even rarer as a cause of spondylodiscitis. We believe our patient contracted the germ in the perioperative setting since she was completely asymptomatic before the surgery. The most probable sources of the rod are the ultrasound gel used to diagnose the cholecystitis or the fluid used during the cholecystectomy, which probably led to transient bacteremia and subsequent bone and intervertebral disc infection.

In conclusion, to our knowledge, this is the first case of lumbar osteomyelitis postcholecystectomy in a healthy young female adult and the first case of* B. cepacia* osteomyelitis in the Middle East. Caution must be used with fluids that may be soiled and used in the health care setting. A high index of suspicion must be kept for perioperative spondylodiscitis, especially with the high prevalence of lower back pain, in order not to delay treatment and avoid potentially life threatening complications.
